# Measuring the Mechanical Properties of Insulin: A Potential Solution to Overcoming the Challenges of Real-Time, Point-of-Care Insulin Sensing

**DOI:** 10.1177/19322968251331072

**Published:** 2025-04-09

**Authors:** Emily Young, Stefanie Gutschmidt, J. Geoffrey Chase

**Affiliations:** 1 Department of Mechanical Engineering, University of Canterbury, Christchurch, New Zealand

**Keywords:** diabetes, insulin detection, micro-electro-mechanical-systems, point-of-care

## Abstract

It is well established real-time, point-of-care capabilities for insulin sensing would provide valuable insight to enhance diabetes management and care in the human body. However, such suitable technology has not yet been developed or commercialized. While not comprehensive, this commentary provides a concise summary of the motivation and challenges of developing real-time, point-of-care insulin sensing technology and offers some comments on current approaches. This short research analysis presents a new perspective on the problem and introduces a future potential solution via measuring the mechanical properties of insulin and discusses the challenges foreseen in the feasibility of this proposed solution.

## Introduction: Why Is It Desirable to Sense Insulin in Real Time and at the Point-of-Care?

There is no question that blood glucose measurements are of great value for the management and treatment of patients with diabetes, particularly for those with insulin dependent diabetes such as type 1. Insulin regimens/therapy are adjusted to optimize the control of glucose, with real-time, point-of-care (POC) blood glucose measurements being a factor in informing the patients about the optimal insulin dose. Whether these are obtained discretely, or via the far more increasingly seen continuous glucose monitors (CGMs), the use of glucose measurements is critical to safe insulin dosing to minimize the risk of hypoglycemia while restricting glucose excursions.

One key safety factor for insulin dosing is the estimate of insulin on board (IOB) from current, ongoing basal dosing to prior bolus insulin which might still be in circulation. Currently, IOB can be estimated by insulin action and decay plots^1^ and in model-based closed loop (artificial pancreas) or semi-closed loop insulin delivery systems.^2^ These estimates can be used in closed loop control or care protocols to help avoid insulin overloading or “bolus stacking” leading to eventual, unintended hypoglycemia. However, they are estimates, as the kinetics, use and clearance of insulin is highly dependent on many unknown factors varying between individuals, as well as over time, such as insulin sensitivity and metabolism, insulin losses and/or transport rates in the subcutis.^3-5^

Unlike blood glucose, blood insulin cannot currently be measured in real time and at POC. Thus, a direct measure would allow safer glycemic control from more comprehensive information and some added potential insight into insulin utilization, clearance, and other losses.^6,7^ Point-of-care testing is defined as “*the analysis of clinical specimens outside the traditional laboratory, near to or at the site of patient care*” (eg, at a patient’s bedside, at home).^8^ However, it should be noted that any first technology developed will likely be for use at a clinical care facility (eg, hospital). The intended aim of a first technology for POC insulin testing is to guide a clinical insulin dosing decision, for example, to decide if a post-meal correction bolus should be administered. For this purpose, the sensor does not need to achieve the 1.4 pmol/L detection capabilities of traditional laboratory immunoassays,^9^ but rather indicate insulin concentration within a minimum of, for example, three detection bands (low/medium/high insulin concentration) and with 20% to 30% accuracy.

### What Are the Current Methods of Insulin Sensing?

Current insulin assays are based on immunoassay (eg, enzyme-linked immunosorbent assay (ELISA)) and analytical chemistry methods (eg, liquid chromatography), but are laboratory based and do not provide instantaneous results. Sensing can take up to 4 hours using immunoassays and approximately 1 hour using analytical chemistry methods,^10^ presuming no time delay in samples being sent to a suitable lab and results being returned. Noting rapid-acting bolus insulin generally reaches peak concentration within 20 to 60 minutes of administration,^11^ such methods are not suitable for informing management of blood glucose levels.

### What Are the Challenges in Achieving Real-Time, Point-of-Care Insulin Sensing?

The detection of insulin presents several well documented and discussed challenges.^10^ These can be briefly summarized:

1. **Reactivity**: Insulin, unlike glucose, cannot be measured using a relatively simple enzymatic oxidation reaction (and instantaneously, within 10 seconds).^12^ To this extent, insulin is nonreactive, hence the need for immunoassay and analytical chemistry methods for traditional detection.
2. **Sensitivity**: The low absolute physiological concentration of insulin in blood volume presents a significant challenge in achieving insulin sensing, with blood insulin concentration of the pico-mole per liter magnitude.^13,14^ This equates to 10^−15^ moles in a milli-liter blood sample for example. By comparison, blood glucose concentration is of the milli-mole per liter magnitude,^11^ equating to 10^−6^ moles for a milli-liter blood sample. Thus, a high sensitivity sensing method is required to measure insulin.
3. **Selectivity**: As is for the detection of any other analyte, suitable selectivity is required for the detection of insulin. This could be achieved by isolating the insulin in the sample prior to sensing, for example, blood insulin bound by a functionalized sensor.

Implementation of a real-time, POC solution presents additional challenges, these include:

4. **Sampling and sample processing**: Adequate selectivity of insulin needs to be achieved within the limits of POC capabilities, and for sampling and sample processing to achieve this without laboratory processes and equipment.
5. **Operating conditions**: The increased sensitivity provided by operating conditions, such as sensing in a vacuum or in cryogenic temperatures, cannot be utilized in a POC solution.
6. **Detection time**: Not only should sensing be instantaneous, but also any associated sample processing.

### What Are the Current Approaches Toward Achieving Real-Time, Point-of-Care Insulin Sensing?

A current common approach toward achieving real-time, POC insulin detection is the sensing of insulin bound by antibodies via electrochemical sensing. Works featuring this means of detection analyze a variety of modified electrodes and sensing methods.^15-18^ Instead of the typical electrochemical sensing approach, Kahanovitz et al^19^ developed a microfluidic platform to measure antibody bound insulin in subcutaneous interstitial fluid using fluorescence. The total detection time was unclear. However, the most significant limitation of this method was the limit of detection (~15 μIU/mL for regular human insulin and insulin lispro, and ~25 μIU/mL for insulin aspart), which was not low enough to detect the interstitial fluid basal insulin concentrations in the range expected for those with type 1 diabetes (4-15 μIU/mL).

Using electrochemical sensing, particularly promising developments have been made by Vargas et al^16,20^ with their near real-time, POC insulin sensing platform. This sensor has displayed sufficient accuracy in comparison to gold standard ELISA results in initial clinical studies with characteristics suitable for POC implementation, such as scalable manufacturing, cost, and storage. However, due to the incubation time for insulin-antibody binding to isolate insulin from the blood, detection required 30 minutes (near real time). They comment, “*alternative technologies that do not require enzymatic labelling for analyte detection will likely be required*” to achieve real-time insulin monitoring.^20^ For example, aptamers could be used to isolate insulin instead of antibodies.^21^

### Using Mechanical Properties of Insulin to Overcome Challenges of Real-Time, Point-of-Care Insulin Sensing

**Figure 1.**
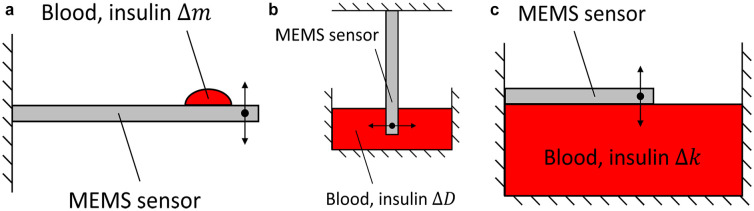
Schematics of mechanics-based insulin sensor concepts (not to scale). (a) Mass sensor. (b) Viscosity sensor. (c) Surface tension sensor.

An entirely mechanics-based approach to real-time, POC insulin sensing involves directly sensing the mechanical properties of insulin in a sample (eg, blood). For example, insulin mass and viscosity properties could be detected. This solution would utilize micro-electro-mechanical-systems (MEMS) technology and sensing concepts used in other applications to achieve high sensitivity. Sensing itself can be instantaneous due to the kilo-Hertz scale natural frequencies of MEMS sensors, corresponding to time in the range of 10^−4^ to 10^−6^ seconds. Examples of mechanics-based insulin sensor concepts based on existing technology are illustrated in Figure 1.

Khater et al^22^ employed electrostatic MEMS sensing to detect ethanol vapor in dry nitrogen gas. They experimentally detected an equivalent mass of the pico-gram scale in seconds. The concept uses the static deflection of the sensor. However, other examples of MEMS sensing utilize dynamic means.^23-26^

Micro-electro-mechanical-system viscosity sensing has previously been applied to glucose detection for CGM.^27^ As the experimental glucose concentration increased, the decrease in peak vibration amplitude (and resonance frequency) correlated with an increase in vibrational damping due to increased solution viscosity. Sensing was achieved in approximately 3 minutes.

Park et al^28,29^ achieved near real-time sensing of insulin by using surface tension, with the sensor resonating dynamically on the air-liquid interface. Their sensing approach required approximately 30 minutes due to insulin-antibody binding.

Insulin-antibody binding via a functionalized sensor is a common method of achieving insulin selectivity for sensing, but it is time consuming (as Vargas et al commented)^20^ and comes with the risk of interferents.^30^ A purely physics-based approach could be utilized as an alternative to functionalization, reducing both the time cost and possibility of cross-reactivity. Microfluidic means^31^ and/or flow cytometry^32^ can be employed to filter the sample and sort molecules by size, such that selectivity is achieved by excluding molecules not similar in size to insulin for sensing. Calibration should provide reasonable exclusion in addition to these measures. Bao et al^33^ previously designed a MEMS sensor achieving selectivity using a similar approach, experimentally capturing particles of the desired size in the filter with success. In the absence of functionalization, the sensor featured a micro-channel with pillars to filter and capture particles for detection in a stream of air. The added mass of the accumulated particles then resulted in a measurable decrease in the sensor’s resonant frequency.

**Figure 2.**
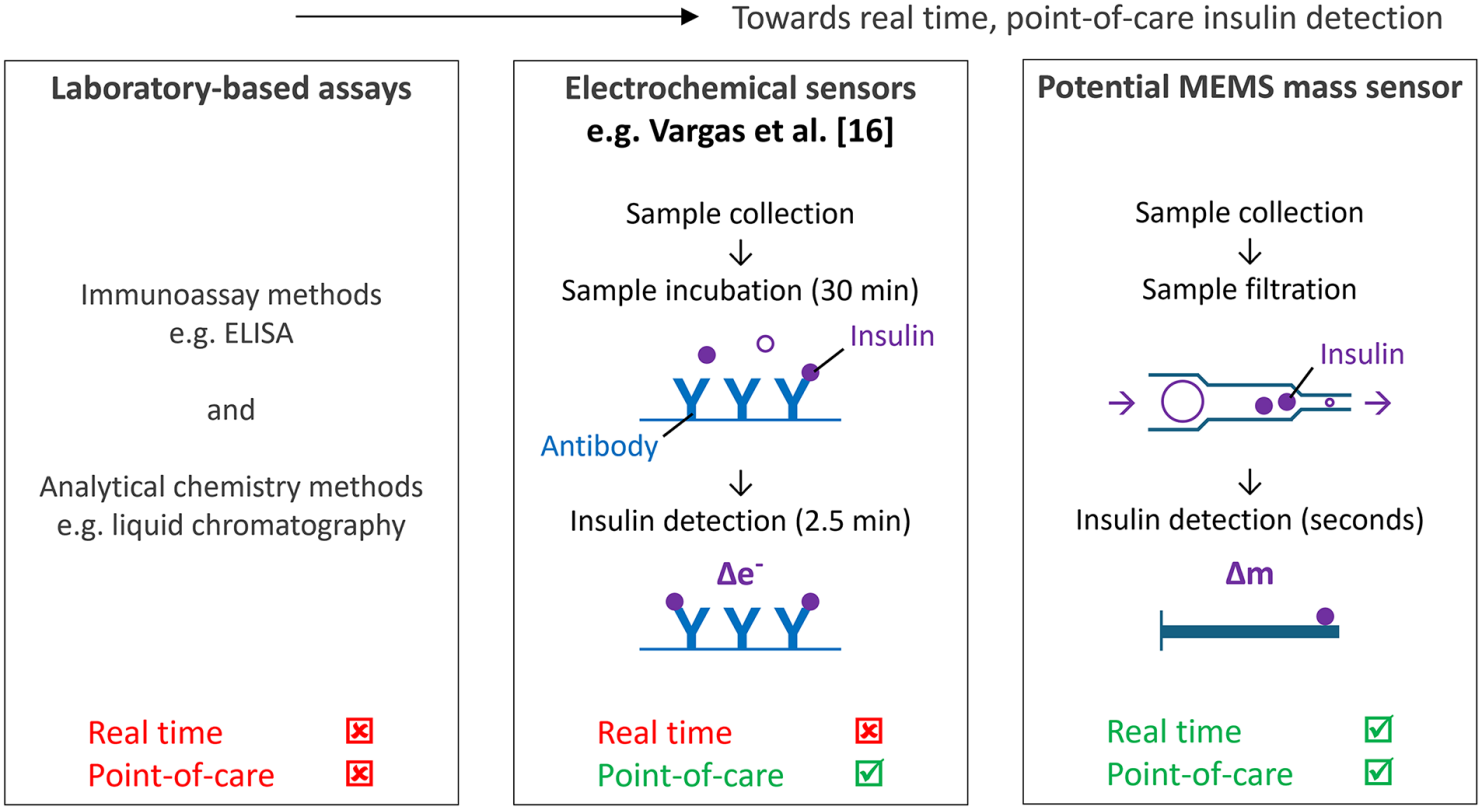
Summary of the development toward real-time, point-of-care insulin detection (schematics not to scale).

Figure 2 summarizes the progression toward achieving real-time, POC insulin detection, including current laboratory-based methods of insulin sensing (“What Are the Current Methods of Insulin Sensing?”), current electrochemical insulin sensing technology (“What Are the Current Approaches Toward Achieving Real-Time, Point-of-Care Insulin Sensing”), and the proposed mechanics-based approach to real-time, POC insulin sensing.

### What Are the Challenges and Requirements of Sensing Insulin’s Mechanical Properties?

Equivalent insulin mass detection ranges have been established for physiological plasma insulin (Table 1) with an input blood volume of 1 to 2 μL obtained using finger prick capillary blood sampling.^34,35^

**Table 1.** Summary of Biological Sensor Parameters for Insulin Detection via Mechanical Properties.

Parameter		Total range	Detection range			Units
			Low	Medium	High	
Physiological plasma insulin		5-200	5-70	70-135	135-200	μIU/mL
		30-1200	30-420	420-810	810-1200	pmol/L
Input blood	Volume^34,35^	1-2				μL
	Diameter^34^	1.24-1.96				mm
Mass of plasma insulin	For 2μL blood, 0.4 μL plasma^a^	0.0694-2.78	0.0694-0.972	0.972-1.87	1.87-2.78	pg
Viscosity of plasma^36^		1.0-1.4				mPa s

a Using a conservative plasma yield of 20% of input blood volume after separation.^37^

#### Viscosity detection

Whole blood is a non-Newtonian fluid, however plasma is considered a Newtonian fluid and its viscosity independent of the shear rate.^38^ Hence, plasma can be treated as a purely viscous fluid, unlike viscoelastic non-Newtonian fluids with both viscous and elastic behavior.

Mellinghoff et al^36^ found that plasma viscosity for patients with type 1 diabetes was in the range 1.0 to 1.4 mPa s (Table 1). Adams et al^39^ later documented the viscosity of insulin analogues themselves (1.064 mPa s for IGlu, to 1.146 mPa s for IBov). However, there is no available data explicitly detailing the relationship between plasma insulin concentration and plasma viscosity. Hence, viscosity parameters for insulin detection could not be determined.

In their application of MEMS viscosity sensing for blood glucose detection, Huang et al^27^ achieved sensitivity of the same scale as the sensor (micro). This indicates that viscosity sensing may not be able to bridge multiple orders of magnitude between the sensor and viscosity to be detected. A nano-scale sensor can be immersed in an input blood sample of 2 μL for viscosity sensing. Hence, it is unlikely that viscosity sensing is feasible if the change in viscosity to be detected is not of the same scale as the sensor (nano).

#### Mass detection

For an input blood sample of 2 μL, the equivalent mass of plasma insulin requires detection of the pico-gram scale (Table 1). A milli-meter scale sensor is also required to accommodate the 2 μL input blood sample with a 1.96 mm diameter. Hence, pico-gram mass detection is to be achieved using a milli-meter scale sensor—a difference of nine orders of magnitude.

It is common to bridge a six order of magnitude gap to sense pico-gram masses using MEMS micro-scale sensors in ambient conditions, utilizing both static^22^ and dynamic^23-26,40^ nonlinear methods. However, achieving sensing for greater relative differences is more challenging, especially for a POC solution.

Chaste et al^41^ and Yang et al^42^ were able to bridge 15 order of magnitude sample-sensor differences to sense yocto-gram and zepto-gram masses respectively. Both achieved sensing experimentally but undertaken in a vacuum. Operation in a vacuum is not suitable for a POC application, despite its capability to enhance sensitivity.

The aforementioned works discuss micro/nano-scale sensors, regardless of sensing method. So, the question is not only can the nine order of magnitude difference be bridged, but also can this sensitivity be achieved for a POC milli-scale sensor?

This challenge in achieving adequate sensitivity can be approached from two sides, to

1. increase sample concentration (eg, via adsorption), and
2. increase sensor sensitivity (via sensor design and sensing method).

Increasing the sample concentration would allow a sample of smaller volume and higher concentration to be tested, compared to the initial input sample (2 μL blood). Consequently, this could enable the sensor scale to be decreased from milli to micro. Concentrating the sample also has the potential to remove the need to functionalize the sensor (eg, with antibodies or aptamers) by way of binding the insulin. Insulin adsorption is usually clinically undesirable but could be exploited for this purpose as a possible method to achieve sample concentration and selectivity. Otherwise, an approach similar to Bao et al’s^33^ micro-pillar filter could be applied for insulin selectivity.

Of course, the sensor design and sensing method are inherently key in bridging the sensitivity gap. Aspects of sensor design such as geometric features (eg, nonuniform beam width, thickness) and configuration (eg, cantilever, clamped-clamped beam, arrays) can increase sensitivity. Similarly, there are several different sensing methods that can be applied utilizing MEMS (static/dynamic, linear/nonlinear) as discussed previously.

Future work looks to determine how many orders of magnitude can be bridged from both sample and sensor sides of the problem, first independently and then together.

## Outlook

Measuring the mechanical properties of insulin provides a potential solution to overcoming the challenges of real-time, point-of-care insulin sensing. Analysis is to be undertaken to determine the feasibility of the mass sensing approach and if the required sensitivity and selectivity can be achieved theoretically, experimentally, and commercially. It is recognized this approach is a future concept, 5+ years of development away, but demonstrates a new approach to insulin sensing.

## References

[1] Zisser H Robinson L Bevier W , et al. Bolus calculator: a review of four “smart” insulin pumps. Diabetes Technol Ther. 2008;10(6):441-444. doi:10.1089/dia.2007.0284. 19049372

[2] Lee H Buckingham BA Wilson DM Bequette BW . A closed-loop artificial pancreas using model predictive control and a sliding meal size estimator. J Diabetes Sci Technol. 2009;3(5):1082-1090. doi:10.1177/193229680900300511. 20144421 PMC2769914

[3] Polidori DC Bergman RN Chung ST Sumner AE . Hepatic and extrahepatic insulin clearance are differentially regulated: results from a novel model-based analysis of intravenous glucose tolerance data. Diabetes. 2016;65(6):1556-1564. doi:10.2337/db15-1373. 26993071 PMC4878413

[4] Najjar SM Perdomo G . Hepatic insulin clearance: mechanism and physiology. Physiology. 2019;34(3):198-215. doi:10.1152/physiol.00048.2018. 30968756 PMC6734066

[5] McHugh AD Chase JG Knopp JL , et al. The impact of exogenous insulin input on calculating hepatic clearance parameters. J Diabetes Sci Technol. 2021;16(4):945-954. doi:10.1177/1932296820986878. 33478257 PMC9264438

[6] Knopp JL Hardy AR Vergeer S Chase JG . Modelling insulin adsorption in intravenous infusion sets in the intensive care unit. IFAC J Syst Control. 2019;8:100042. doi:10.1016/j.ifacsc.2019.100042.

[7] Knopp JL Chase JG . Clinical recommendations for managing the impact of insulin adsorptive loss in hospital and diabetes care. J Diabetes Sci Technol. 2020;15(4):874-884. doi:10.1177/1932296820915875. 32329372 PMC8258516

[8] New Zealand Point-of-Care Testing Advisory Group. New Zealand Best Practice Guidelines for Point-of-Care Testing 2022. 2022. https://irp.cdn-website.com/102112c1/files/uploaded/2022%20NZPOCTAG%20Guidelines.pdf. Accessed April 1, 2025.

[9] Roche Diagnostics. Elecsys insulin method sheet. 2024. https://elabdoc-prod.roche.com/eLD/web/global/en/products/CPS_000491. Accessed January 16, 2025.

[10] Soffe R Nock V Chase JG . Towards point-of-care insulin detection. ACS Sensors. 2019;4(1):3-19. doi:10.1021/acssensors.8b01253. 30525462

[11] Holt RIG Cockram C Flyvbjerg A Goldstein BJ . Textbook of Diabetes. Hoboken, NJ: John Wiley & Sons, Incorporated; 2017.

[12] Yoo EH Lee SY . Glucose biosensors: an overview of use in clinical practice. Sensors (Basel). 2010;10(5):4558-4576. doi:10.3390/s100504558. 22399892 PMC3292132

[13] Vargas E Nandhakumar P Ding S Saha T Wang J . Insulin detection in diabetes mellitus: challenges and new prospects. Nat Rev Endocrinol. 2023;19(8):487-495. doi:10.1038/s41574-023-00842-3. 37217746 PMC10202074

[14] Psoma SD Kanthou C . Wearable insulin biosensors for diabetes management: advances and challenges. Biosensors (Basel). 2023;13(7):719. doi:10.3390/bios13070719. 37504117 PMC10377143

[15] Wardani NI Kangkamano T Wannapob R Kanatharana P Thavarungkul P Limbut W . Electrochemical sensor based on molecularly imprinted polymer cryogel and multiwalled carbon nanotubes for direct insulin detection. Talanta. 2023;254:124137. doi:10.1016/j.talanta.2022.124137. 36463801

[bibr16-19322968251331072] Vargas E Aiello EM Pinsker JE , et al. Development of a novel insulin sensor for clinical decision-making. J Diabetes Sci Technol. 2022;17(4):1029-1037. doi:10.1177/19322968211071132. 35043720 PMC10347992

[bibr17-19322968251331072] Khanwalker M Fujita R Lee J , et al. Development of a POCT type insulin sensor employing anti-insulin single chain variable fragment based on faradaic electrochemical impedance spectroscopy under single frequency measurement. Biosens Bioelectron. 2022;200:113901. doi:10.1016/j.bios.2021.113901. 34968857

[18] Park YM Choi YS Lee H-R , et al. Flexible and highly ordered nanopillar electrochemical sensor for sensitive insulin evaluation. Biosens Bioelectron. 2020;161:112252. doi:10.1016/j.bios.2020.112252. 32442107

[19] Kahanovitz L Seker E Marks RS Yarmush ML Konry T Russell SJ . Development of a microsphere-based system to facilitate real-time insulin monitoring. J Diabetes Sci Technol. 2015;10(3):689-696. doi:10.1177/1932296815625081. PMC5038538 26721524

[20] Aiello EM Pinsker JE Vargas E , et al. Clinical evaluation of a novel insulin immunosensor. J Diabetes Sci Technol. 2022;17(4):1038-1048. doi:10.1177/19322968221074406. 35118893 PMC10347985

[21] Luong A-D Roy I Malhotra BD Luong JHT . Analytical and biosensing platforms for insulin: a review. Sens Actuators Rep. 2021;3:100028. doi:10.1016/j.snr.2021.100028.

[22] Khater ME Al- Ghamdi M Park S , et al. Binary MEMS gas sensors. J Micromech Microeng. 2014;24(6):065007. doi:10.1088/0960-1317/24/6/065007.

[23] Rabenimanana T Najar F Ghommem M Walter V Kacem N . On the equivalence between mass perturbation and DC voltage bias in coupled MEMS resonators: theoretical and experimental investigation. J Appl Phys. 2022;132(2):024502. doi:10.1063/5.0097377.

[24] Li L Liu H Shao M Ma C . A novel frequency stabilization approach for mass detection in nonlinear mechanically coupled resonant sensors. Micromachines. 2021;12(2):178. doi:10.3390/mi12020178. 33670263 PMC7917976

[25] Meesala VC Hajj MR Abdel- Rahman E . Bifurcation-based MEMS mass sensors. Int J Mech Sci. 2020;180:105705. doi:10.1016/j.ijmecsci.2020.105705.

[26] Zhang W Turner KL . Application of parametric resonance amplification in a single-crystal silicon micro-oscillator based mass sensor. Sens Actuators A: Phys. 2005;122(1):23-30. doi:10.1016/j.sna.2004.12.033.

[27] Huang X Li S Schultz JS Wang Q Lin Q . A MEMS affinity glucose sensor using a biocompatible glucose-responsive polymer. Sens Actuators B: Chem. 2009;140(2):603-609. doi:10.1016/j.snb.2009.04.065. 24511207 PMC3916006

[28] Park J Nishida S Lambert P Kawakatsu H Fujita H . High-resolution cantilever biosensor resonating at air–liquid in a microchannel. Lab Chip. 2011;11(24):4187-4193. doi:10.1039/C1LC20608G. 22038280

[29] Park J Karsten SL Nishida S Kawakatsu H Fujita H . Application of a new microcantilever biosensor resonating at the air–liquid interface for direct insulin detection and continuous monitoring of enzymatic reactions. Lab Chip. 2012;12(20):4115-4119. doi:10.1039/C2LC40232G. 22847153

[30] Tate J Ward G . Interferences in immunoassay. Clin Biochem Rev. 2004;25(2):105-120. 18458713 PMC1904417

[31] Kwon J-Y Kim T Kim J Cho Y . Particle focusing under Newtonian and viscoelastic flow in a straight rhombic microchannel. Micromachines. 2020;11(11):998. 33187390 10.3390/mi11110998 PMC7696856

[32] van Gaal EVB Spierenburg G Hennink WE Crommelin DJA Mastrobattista E . Flow cytometry for rapid size determination and sorting of nucleic acid containing nanoparticles in biological fluids. J Contr Release. 2010;141(3):328-338. doi:10.1016/j.jconrel.2009.09.009. 19778559

[33] Bao Y Cai S Yu H Xu T Xu P Li X . A resonant cantilever based particle sensor with particle-size selection function. J Micromech Microeng. 2018;28(8):085019. doi:10.1088/1361-6439/aabdac.

[34] Heinemann L Boecker D . Lancing: Quo Vadis? J Diabetes Sci Technol. 2011;5(4):966-981. doi:10.1177/193229681100500420. 21880240 PMC3192604

[35] Grady M Pineau M Pynes MK Katz LB Ginsberg B . A clinical evaluation of routine blood sampling practices in patients with diabetes: impact on fingerstick blood volume and pain. J Diabetes Sci Technol. 2014;8(4):691-698. doi:10.1177/1932296814533172. 24876439 PMC4764211

[36] Mellinghoff AC Reininger AJ Wurzinger LJ Landgraf R Hepp KD . Influence of glycemic control on viscosity and density of plasma and whole blood in type-1 diabetic patients. Diabetes Res Clin Pract. 1996;33(2):75-82. doi:10.1016/0168-8227(96)01279-X. 8879961

[37] Gonzalez-Suarez AM Stybayeva G Carey WA Revzin A . Automated microfluidic system with active mixing enables rapid analysis of biomarkers in 5 μL of whole blood. Analyt Chem. 2022;94(27):9706-9714. doi:10.1021/acs.analchem.2c01139. 35604796

[38] Levick JR . Chapter 7: haemodynamics: pressure, flow and resistance. In Levick JR , ed. An Introduction to Cardiovascular Physiology. Oxford, England: Butterworth-Heinemann; 1991:90-116.

[39] Adams GG Meal A Morgan PS , et al. Characterisation of insulin analogues therapeutically available to patients. PLoS ONE. 2018;13(3):e0195010. doi:10.1371/journal.pone.0195010. PMC5875863 29596514

[40] Alkaddour M Ghommem M Najar F . Nonlinear analysis and effectiveness of weakly coupled microbeams for mass sensing applications. Nonlinear Dyn. 2021;104(1):383-397. doi:10.1007/s11071-021-06298-2.

[41] Chaste J Eichler A Moser J Ceballos G Rurali R Bachtold A . A nanomechanical mass sensor with yoctogram resolution. Nat Nanotechnol. 2012;7(5):301-304. doi:10.1038/nnano.2012.42. 22466856

[42] Yang YT Callegari C Feng XL Ekinci KL Roukes ML . Zeptogram-scale nanomechanical mass sensing. Nano Lett. 2006;6(4):583-586. doi:10.1021/nl052134m. 16608248

